# Data-driven synthetic microbes for sustainable future

**DOI:** 10.1038/s41540-025-00556-4

**Published:** 2025-07-07

**Authors:** Iqra Mariam, Ulrika Rova, Paul Christakopoulos, Leonidas Matsakas, Alok Patel

**Affiliations:** Biochemical Process Engineering, Division of Chemical Engineering, Department of Civil, Environmental, and Natural Resources Engineering, Luleå University of Technology, SE-971 87 Luleå, Sweden

**Keywords:** Synthetic biology, Computer modelling, Genomic engineering

## Abstract

The escalating global environmental crisis demands transformative biotechnological solutions that are both sustainable and scalable. This perspective advocates Data-Driven Synthetic Microbes (DDSM); engineered microorganisms designed through integrating omics, machine learning, and systems biology to tackle challenges like PFAS degradation, greenhouse gas mitigation, and sustainable biomanufacturing. DDSMs offer a rational framework for developing robust microbial systems, reshaping the future of synthetic biology toward environmental resilience and circular bioeconomy.

## Introduction

Human activities have had a profound and catastrophic impact on the environment, leading to the depletion of natural resources, increasing greenhouse gas (GHG) emissions, and the pervasive introduction of microplastics in the ecosystem^1^. As we reach the decade milestone of the 15-year timeline set by the 2030 Agenda for Sustainable Development, adopted by all United Nations Member States in 2015, significant challenges remain in achieving the ambitious Sustainable Development Goals (SDGs)^2^. Despite progress in some areas, the urgency to accelerate transformative actions has never been greater.

As Newton’s third law states “*For every action there is an equal and opposite reaction,*” nature has found its course to respond to these challenges, with microbes emerging as front-line warriors in addressing these threats^3^. Microbes play an indispensable role in Earth’s environmental system, facilitating the biogeochemical cycles, capturing and transforming GHG emissions and regulating carbon flow in the ecosystem^4^. From producing alternative fuels to degrading pollutants like plastic, microbes offer innovative solutions to a range of environmental concerns. Their inherent metabolic capabilities offer promising solutions for ecosystem restoration, food production, transportation, and even the development of eco-friendly building materials^5^.

However, the conventional approach to leveraging microbes for such solutions involves labor-intensive and time-consuming screening of microbial diversity. These microscopic organisms often rewire their metabolism; by incorporating genome-level aberrations to cope and thrive in adverse environments^6^. While such evolutionary adaptations are advantageous in nature, they frequently misalign with human applications, such as mitigating environmental impacts or optimizing resource efficiency. To overcome these limitations, microbes can be reprogrammed using genetic engineering or by adaptive laboratory evolution (ALE) under a constrained environment. Synthetic biology bridges this gap by enabling modifications to microbial genetic code, tailoring them for specific sustainable applications^7^. The field of synthetic biology has advanced largely in the past decade; from introducing single gene edits to creating synthetic genomes, organelles and even engineered microbial communities^8^. While the field has largely uplifted several sustainable processes, the complexity of biological systems, unpredictable outcomes and inefficient resilience of synthetic microbes are major hurdles to this approach.

In parallel, omics technologies and computational biology have revolutionized how we study biological systems by providing large-scale datasets spanning all levels, from genes to metabolites. Current omics datasets encompass organisms ranging from the smallest bacterium, *Mycoplasma*, to the largest multicellular eukaryote, *Balaenoptera musculus* (blue whale)^9,10^. Combining these omics datasets provides a holistic understanding of the microbe’s metabolism and its interaction with environmental factors, capturing intricate interactions across genes, proteins and metabolites. System biology leverages these datasets as its foundation, employing mathematical modeling, computational simulations and machine learning (ML) algorithms to transform static information into a dynamic predictive framework of microbial function and behavior^11^.

**Fig. 1:**
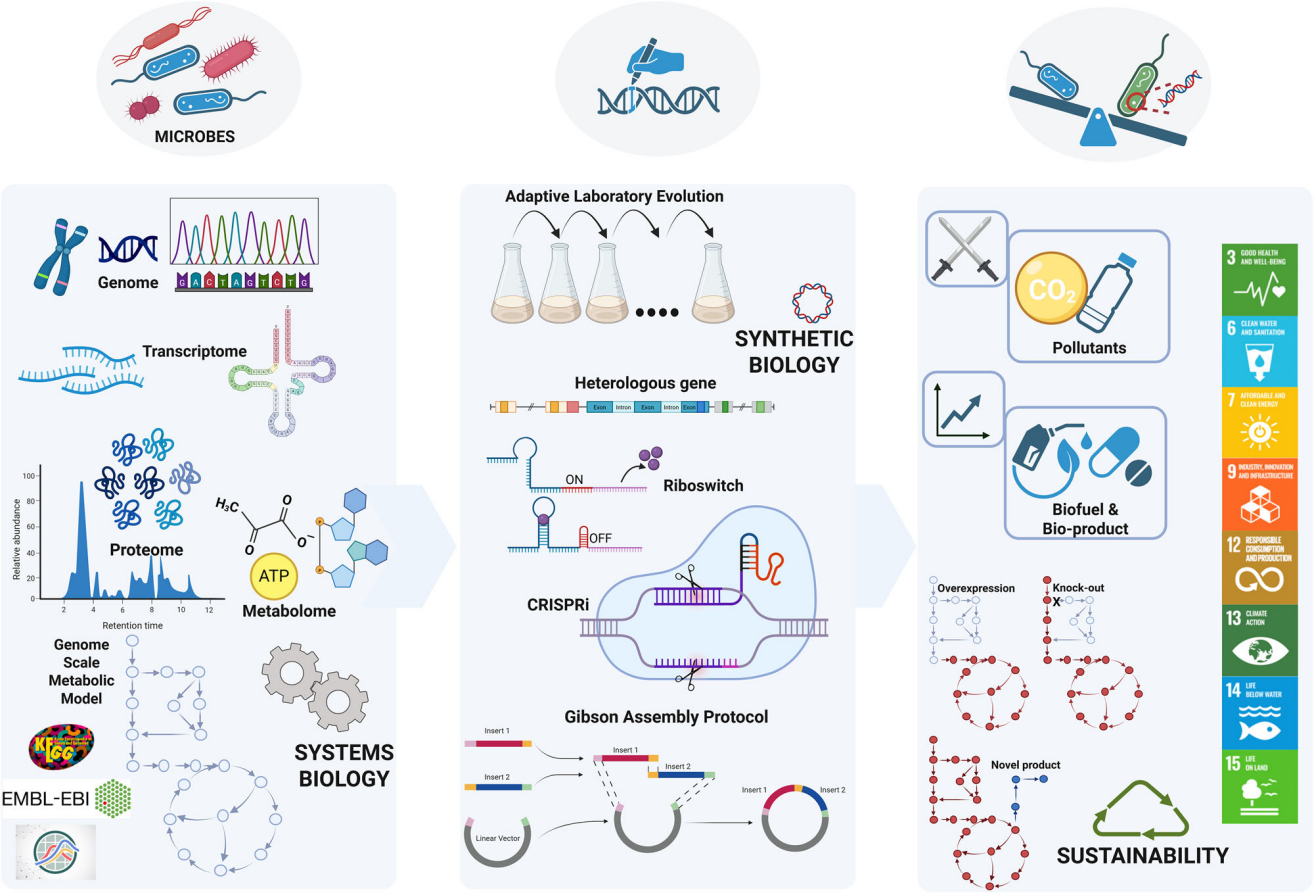
Integrating system biology and synthetic biology tools for creating DDSM for offering sustainable solutions. System biology can leverage information from databases, such as KEGG, EMBL-EBI, generate sequencing data: genomics, transcriptomics, metabolomics & proteomics and generate metabolic models (BioModels). The synthetic biology tools such as Adaptive Laboratory Evolution, CRISPR, riboswitches and Gibson Assembly. Created in BioRender. Mariam, I. (2025) https://BioRender.com/8ieg6au.

Together, omics and systems biology form a powerful synergy, laying the groundwork for advancement in synthetic biology. For instance, employing genomics, an organism’s genetic makeup can be mapped, bioinformatics approaches such as genome mining can enable the selective identification of novel biosynthetic gene clusters for production of value-added products. The integration of mined genomic data into systems biology frameworks further enhances metabolic network reconstruction, enabling functional characterization and pathway design. Systems biology, leveraging multi-omics datasets, can then be employed to predict thermodynamically feasible metabolic routes and to design non-native metabolic pathways aligned with specific engineering goals^12–14^. These insights, coupled with predictive modeling, enable synthetic biology to precisely design and control metabolic pathways in engineered microbes, optimizing them for specific sustainable applications (Fig. 1). ML and predictive modeling approaches accelerate the microbial engineering process by simulating the physiology of engineered microbes before laboratory experiments, minimizing trial and error. In this context, the DBTL cycle (Design-Build-Test-Learn (DBTL)) becomes essential, enabling rapid iteration of the synthetic biology workflow by designing metabolic pathways, building microbial systems, testing their performance, and refining designs based on experimental feedback^15^.

In this perspective, we argue that microbes, reprogrammed through data-driven synthetic methodologies, have the potential to significantly accelerate the journey toward sustainability by offering innovative solutions to meet the SDGs within the forecasted time frame. The article explores how the convergence of systems biology and synthetic biology provides a robust framework for designing microbes to address critical challenges in attaining sustainability. Key obstacles in the field, such as the integration of diverse and complex datasets, accurate modeling of biological systems, and overcoming issues of scalability and real-world variability, are explored as critical areas requiring attention to unlock the full potential of these methods. Furthermore, we propose that the incorporation of digital twins’ technology could lead to breakthroughs in synthetic biology, bioengineering, and materials science, driving transformative innovation in sustainable development.

## Big Data in biology

As of 2024, it has been 47 years since the first complete DNA sequencing of the bacteriophage ϕX174 genome by Sanger et al.^16^. Compared to the 5386 bases genome of ϕX174, advancements in sequencing technologies have revolutionized the genomics field and enabled the study of huge genomes, such as that of *Tmesipteris oblanceolata*, which contains 160 billion base pairs, eclipsing the human genome by 50 times^17^. In addition to genomics datasets, mass spectrometry and high throughput molecular biology technologies generate piles of omics datasets categorized into proteome, metabolome, phenome, interactome etc^18^. Such “Big Data” encompasses both structured and unstructured information, which is difficult to store, process and interpret. To consolidate and organize such complex datasets, several databases are continuously developed and updated to accommodate growing data needs. Such databases are significant for the scientific community, contributing to data-sharing, open access and conversion of raw information into valuable knowledge^19^. Ma et al. curated a catalog for such biological databases; Database Commons which contained 5825 biological databases in 2023 and has increased 19.2% till this date covering 1728 species^20^.

Currently, the well-known publicly accessible biological repositories EMBL (www.ebi.ac.uk/) store approximately 100 petabytes (10^15^ bytes) of biological raw data. EMBL data resources are well categorized into 54 data resources that span from the nucleotide sequences database (European Nucleotide Archive (ENA)) to the large archive for mathematical models of biological and biomedical systems (BioModels)^21,22^. These datasets provide insights into genetic architecture, protein interactions, and metabolic pathways, which can be harnessed for optimizing biological systems to drive sustainable solutions. In the context of bioremediation, omics datasets have identified key proteins and metabolic pathways responsible for the degradation of oil spills, hydrocarbons, plastic and even per- and polyfluoroalkyl substances (PFAS). 16S rRNA sequencing has identified several bacterial strains that are capable of degrading PFAS, such as *Pseudomonas plecoglossicida* 2.4-D and *Labrys portucalensis* F11^23,24^. Further, coupling 16S rRNA identification with metabolomics for soil samples collected near a fluoropolymer production facility has identified the microbial community structure along with relevant metabolic pathways that aid in the degradation of these forever chemicals. Another study by Dong et al., identified key microorganisms such as *Variovorax, Rhodococcus*, and *Cupriavidus* using 16S rRNA gene sequencing. They employed PICRUSt2 for functional prediction, inferring potential genes involved in PFAS biotransformation from 16S rRNA amplicon sequencing data, such as 6-oxocyclohex-1-ene-carbonyl-CoA hydrolase gene and cyclohexa-1,5-dienecarbonyl-CoA hydratase^25^. Despite these advancements, a comprehensive understanding of the complete degradation pathway of PFAS is still elusive. This knowledge gap is probably attributed to the high proportion of genes identified in omics datasets that often encode proteins of unknown function, especially from uncultured or poorly characterized microbes. This ‘microbial dark matter’ limits our ability to reconstruct complete degradation pathways for pollutants like PFAs. Emerging approaches like culturomics offer promising avenues to isolate and experimentally validate the role of these elusive microbes, complementing metagenomic predictions and enriching existing reference databases^26^. In addition to generating more omics datasets, the development of AI-driven tools like DeepARG & HMD-ARG, which utilizes deep learning to predict antibiotic resistance genes from metagenomic data, highlights the growing potential of ML approaches in functional gene annotation^27,28^. Adapting similar computational frameworks to predict novel catabolic genes involved in PFAS degradation or other environmental pollutant breakdown pathways could significantly enhance our capacity to interpret metagenomic data and accelerate the discovery of functional microbial traits.

## Leveraging computational tools to decode Big Data

The integration of computer science, mathematical modeling, and algorithmic approaches has revolutionized biological data analysis, enabling access to, interpretation of, and insight from massive omics datasets. However, it is important to distinguish between traditional bioinformatics tools and ML methods. For example, NCBI-BLAST (Basic Local Alignment Search Tool) is a widely used heuristic algorithm for sequence alignment and similarity searches; while computationally powerful, it is not considered a ML method, as it does not involve model training or prediction based on data-driven learning^29^. In contrast, ML refers to a class of computational techniques where models are trained on data to learn patterns and make predictions. ML plays a pivotal role in biological data analysis, such as extracting features from complex datasets and enabling predictive modeling. Deep Learning, a subset of ML, underpins tools like AlphaFold, which revolutionized protein structure prediction by learning from vast protein sequence and structure data. Both supervised methods (e.g., neural networks, support vector machines) and unsupervised methods (e.g., principal component analysis, clustering) are widely applied in omics-driven synthetic biology^30^. These approaches facilitate the interpretation of complex datasets, such as identifying key proteins or metabolites responsible for combating pollutants. After the Deepwater Horizon accident in the Gulf of Mexico (2010), omics technologies have been hugely employed for evaluating marine microbial communities that aid the process of petroleum biodegradation. Bacterial communities identified after the spill were enriched in *Oceanospirillaceae, Pseudoalteromonas, Pseudomonas, Vibrio, Acinetobacter, Alteromonas, Colwellia*, and *Cycloclasticus* that are known as obligate hydrocarbonoclastic bacteria^31^. The uptake and metabolic pathway for degradation of such oil pollutants vary from species to species. *Alcanivorax borkumensis*, for instance, forms a spherical biofilm around oil drops, which then expands and deforms^32^. Our group at *Lulea University of Technology, Sweden* has identified the potential of marine thraustochytrid, *Schizochytrium limacinum* SR21 to assimilate up to 120 g of waste cooking oil^33^. Transcriptomics datasets generated for *S. limacinum* SR21 have revealed that degradation initiates outside the cell itself, mediated by serine hydrolases (triacylglycerol acylhydrolases, E.C. 3.1. 1.3)^33,34^. The marine thraustochytrid possesses 20 serine hydrolases, differentially localized across cellular compartments, plasma membrane and secretory pathways. A large-scale omics dataset comprising 418 microbiome and 125 metabolome samples was analyzed to investigate microbial niche differentiation and succession on various biodegradable polymers, including polycaprolactone, polybutylene succinate-co-adipate, polybutylene succinate, polybutylene adipate-co-terephthalate, and poly(3-hydroxybutyrate-co-3-hydroxyhexanoate)^35^. In this case, several ML methods, such as Bayesian Hierarchical Modeling (Generalized Linear Mixed Model) and Association Rule Mining (Apriori Algorithm) were employed for predictive functional analysis and pattern recognition. The study lays a foundation for understanding polymer degradation in the environment, emphasizing the hydrolase gene as the key determinant of microbial community structure specific to each polymer type. Synthetic biology can leverage this information, by engineering microbes or microbial communities that can be designed to efficiently degrade plastics. Additionally, optimizing enzyme efficiency using ML and CRISPR-based protein engineering approaches can further elevate bioremediation.

## Data-driven design of synthetic enzymes through ML

Conventional protein engineering methods involve evaluating amino acid substitutions, chimeric combinations of native protein parts, or directed evolution. Such changes are introduced to improve protein yields, stability, efficiency and optimal activity at challenging temperature or pH. Several modifications are being introduced into AlphaFold, such as TmAlphaFold for predicting structures for membrane proteins and AlphaFold-Multimer for predicting structures for multimeric proteins^36,37^. With the availability of huge databases for both sequences and structures for proteins, ML algorithms can revolutionize the field of protein design. There exist three broad models for the ML-based design of proteins – sequence-based models, sequence-labeled models and structure-based models^38^. Among the two sequence dependent models, the sequence-based model uses an unsupervised algorithm to learn from the pattern of homologous sequences, while the supervised sequence-label model utilizes functional labels (structural domain, motif residues or entire protein) as training datasets to predict protein structure and function^39,40^. For example, convolutional neural networks (CNNs) are widely used in structure label models that can efficiently detect spatial patterns in protein sequences and structural data. Such CNN based model, MutCompute was used to engineer a robust poly (ethylene terephthalate) (PET) hydrolase, that depolymerizes plastic (PET) waste^41^. The mutant FAST-PETase (functional, active, stable and tolerant PETase) shows superior hydrolytic activity and can degrade 51 different thermoformed products completely within 1 week. The MutCompute algorithms utilize 19,000 sequence-based protein structures from PDB, to obtain the probability distribution for the optimal structural fit of all 20 amino acid residues at every position in the protein sequence, conducting an in silico mutational scan. Another ML-based approach, ESMFold; a sequence-to-structure predictor is nearly as accurate as alignment-based methods and is considerably faster^42^. Additionally, generative models, such as variational autoencoders and generative adversarial network algorithms can also be used for de novo protein designs that learn from probability distribution for sequence/structure generations^43,44^. Thus, with this ML-based protein engineering, new-to-nature proteins can be designed, accelerating the development of synthetic enzymes for industrial and pharmaceutical applications.

## Data-driven design of metabolic pathways

Beyond manipulating a single protein, developing a sustainable microbial solution requires understanding the intricate metabolic network, optimizing flux through pathways, and evaluating the feasibility of non-native pathways. By integrating genomic sequencing, transcriptomic analysis, and metabolomic profiling, the entire metabolic network of an organism can be traced. Several ML algorithms, such as kernel or graph-based, unsupervised multi-omics factor analysis and network based OmicsNet are employed for multi-omics data integration and extracting features of interest^45–48^.

The genomic datasets of an organism can be converted into a mathematical model (Genome Scale Metabolic Model; GSMM) that is a stoichiometry-based structural model; a blueprint of microbial metabolism. The constraint-based reconstruction and analysis (COBRA) Toolbox was the first MATLAB software suite developed for the reconstruction of constraint-based models, that are used for quantitative prediction of metabolic phenotype using flux balance analysis (FBA)^49^. The COBRA approach enforces physicochemical, biological, and data-driven constraints to predict the phenotypic outcome for a biological system in a specific condition. These constraints include compartmentalization of reactions within different subcellular spaces, mass balance of metabolites, molecular crowding, which imposes physical constraints on metabolite concentration within a spatial region of a compartment, and thermodynamic feasibility of metabolic reactions. The GSMM model for *Saccharomyces cerevisiae*, a eukaryotic host with several biotechnological applications, has undergone 20 iterations of refinement since the first model iFF708, in 2003^50^. The traditional GSMM is solely stoichiometry-based and thus struggles to reflect dynamic gene expression changes. However, integrating omics data (transcriptomics/proteomics) with constrained GSMMs allows context-specific modeling, improving the simulation of metabolic changes^51^. The consensus GEM for *S. cerevisiae*, Yeast7, was enhanced with enzymatic constraints: kinetic and proteomics data using GECKO toolbox, a technique created in 2017^52,53^. Due to limited experimental k_cat_ data, ML and deep learning models (e.g., DeepEC, DLKcat, TurNup) have been developed to predict enzyme properties^54–56^. DLKcat and TurNup use protein sequences and substrate information to predict k_cat_, with TurNup showing better performance for novel enzymes. When ecGEM predictions deviate from experiments, k_cat_ correction methods adjust values using substitution, scaling, or optimization approaches like PRESTO^57^. On the other hand, mechanistic models models that are built on detailed biochemical and biophysical principles, offer deeper insights than enzyme constrained, often using differential equations to describe the dynamics of molecular interactions and reactions over time^58^.

The current *Yeast9* model is a context-specific model that is curated using proteomics, single-cell transcriptomics and Gibb’s free energy of reactions^59^. Additionally, apart from constraint-based models, kinetic and mechanistic models are often employed for simulation and prediction of phenotypic outcomes. The steady-state assumption made by traditional FBA is not optimal for a rapidly changing environment. This limitation can be addressed by dynamic FBA (dFBA), which iteratively performs FBA over time intervals to capture dynamic metabolic changes. Both static (SOA) and dynamic (DOA) optimization techniques are available for dFBA; where the SOA uses linear programming, while DOA employs non-linear programming for whole-period optimization^60^.

Thus, metabolic flux analysis allows us to establish a link between phenotypic outcomes and underlying metabolic processes, enabling the identification of potential engineering targets for synthetic biology. An in-silico simulation of overexpression/knockout in GSMMs can be performed using algorithms such as OptORF, OptKnock, OptForce, and RobustKnock^61–64^. Further, tools, such as ROOM (regulatory on/off minimization), RELATCH (minimization of relative metabolic change) and MOMA (minimization of metabolic adjustment) can predict the flux distributions in knockout strains^65–67^. Recently, a ML algorithm FluxRETAP was developed at the US Department of Energy Agile BioFoundry, United States for the prediction of hundreds of gene targets within minutes for knockout/overexpression to produce a certain value-added product^68^.

## Advances in synthetic biology

The field of synthetic biology has largely advanced since the discovery of the DNA double helix, enzymes, such as polymerases, ligases and restriction endonuclease. With the advent of chemical methods for synthesis of DNA/nucleotides and polymerase chain reaction has opened avenues to isolate targeted gene or gene fragments, amplify the locus and introduce non-native genes into organisms. Till this date, there has been several developments in the tools and methods for genome editing, and one of the major breakthroughs is Clustered Regularly Interspaced Palindromic Repeats (CRISPRs), and their associated (Cas) nucleases system, which was awarded the Nobel Prize in Chemistry (2020)^69^. CRISPR-Cas9 based gene editing has made significant contributions in revolutionizing agriculture, biomedicine, gene therapy for cancers and other applications in sustainable biology^70–73^. Several variants of RNA-guided nucleases have been developed so far, with diverse characteristics and limitations such as PAM constraints, off-target risks, or immunogenicity^74^. The robust design, specificity and efficiency of CRISPR based genetic editing can benefit from ML models that can predict optimal guide RNA sequences in design and the number of off-targets^75^. Beyond CRISPR and targeted gene-editing, Golden Gate Assembly and Modular Cloning can be employed for construction of multi-gene or multi-gene component constructs using standardized parts such as promoters, terminator, ribosome binding sites (RBS) etc^76,77^. A python based workflow; DnaCouldron, designed by Edinburgh Genome Foundry, allows simulating assembly of large DNA fragments to predict the outcome of final construct and flaws in the assembly^78^. Gibson Assembly complements these methods with its ability to seamlessly join overlapping DNA fragments, making it essential for assembling large or custom genetic elements^79^. Neural networks can be utilized to guide the design of RBS and promoters for controlling gene expression of synthetic constructs^80,81^. For heterologous expression of genes, a major bottleneck is the codon specificity, to address which a deep learning model CodonTransformer was developed that was trained on over 1 million DNA-protein pairs from 164 organisms^82^. Additionally, for biosensors and dynamic genetic circuits, riboswitches and other RNA-based regulators, such toehold switches, provide precise, ligand-responsive control of gene expression^83^. Optogenetic tools such as light-responsive proteins and chemo-optical switches allow spatiotemporal control to use light or chemical cue to regulate gene expression in an organism. For instance, by integrating red light responsive FHY1 and *ΔphyA* photoreceptors, a red light inducible Cre recombinase system (OptoCre-REDMAP) was introduced in *Escherichia coli*^84^. Furthermore, metabolic pathways can be autonomously optimized by reinforcement learning techniques, negating the need for time-consuming trial-and-error experimentation. A commentary section written by Christopher A. Voigt (2020) has discussed six products that are developed using synthetic biology approaches and can be introduced in the market^8^. Several companies like Pando Biosciences, Volta Labs, Synthexo and Amyris currently use artificial intelligence (AI) and synthetic biology to design microorganisms that offer biotechnological solutions^85^.

## Digital twins and synthetic biology

A digital twin is a dynamic, virtual representation of any process that integrates experimental data at different levels, such as genetic makeup, environmental factors, and process parameters, to simulate and predict the phenotypic outcome. Digital twins in synthetic biology allow for iterative design and optimization of engineered microorganisms by creating a closed-loop connection between metabolic models, experimental feedback, and omics data, generation of mutants and experimental parameters of a process^86^. Digital twins can ease the optimization of microbial processes by employing iterative DBTL cycles, real-time monitoring and process adjustments, and minimal trial-and-error experimentation. This enhances efficiency, reduces waste, and ensures the scalability of sustainable bioproduction. This technology heavily relies on high-throughput data acquisition, computational modeling, AI, and automation^87^. Creating a virtual twin is a multi-step process that initiates with the collection of experimental and multi-omics datasets, extracting the specific feature for the phenotype of interest and mapping them into biological pathways. The next step is dynamic/constraint-based modeling using the features from multi-omics datasets and in silico identification of knockouts to increase the yield of the desired product, guiding the re-engineering of the microbial strain. Ultimately, the dynamic models’ predictions are validated experimentally, and a virtual twin is developed that provides deeper insights into the biological process underlying product formation.

Digital twins can offer promising applications in sustainable manufacturing of products and polymers, bioremediation, carbon sequestration, and environmental monitoring; the concept is still far-fetched. With several challenges associated with the implementation of such technology, there is not a single real-time digital twin employed for synthetic biology yet. A useful tool for computer-aided design and simulation of synthetic biological systems, the Infobiotics Workbench (IBW) supports in silico modeling of metabolic processes and gene circuits^88^. However, IBW functions as an offline simulation environment and lacks the essential features of a true digital twin, such as dynamic process manipulation and real-time data integration. As a result, it presents scalability issues due to its limited capacity to adaptively direct biomanufacturing processes under shifting experimental settings. Another bottleneck in implementation of digital twins for synthetic biology is automated high-throughput screening platforms and robotics platforms for evaluating hundreds of synthetic microbial strains to configure high-yielding microbes^89^. Berkeley Lights’ Beacon system, which use optofluidic technology to rapidly screen, track, and choose high-performing cell lines at single-cell resolution, is one encouraging tool in this regard. The Beacon system is an example of the kind of automated experimental feedback loop that might be included into a digital twin framework for real-time optimization of engineered strains based on performance parameters, even though it is not a digital twin in and of itself^90^. In order to create more sustainable biological solutions, researchers can iteratively improve their ideas by fusing automated screening with ML.

## Synthetic microbial communities (SynCom) for sustainability

Naturally, microbes rarely act alone, as they are social, symbiotic, and deeply intertwined, and form complex communities that result in adaptation or survival. Each microscopic microbe plays a pivotal role in an intricate and dynamic biological community process, exchanging metabolites, signals, and genetic information in a choreography. Synthetic microbial communities (SynCom) mimic the cooperative dynamics found in natural microbial consortia, and allow division of labor within specialized strains and improve the overall performance^91^. These engineered communities offer a robust framework for developing resilient, efficient, and adaptable biological systems. Brenner et al., has reviewed the concept of engineering microbial consortia in 2008, followed by which SynCom have been employed for several biosynthetic, bioprocessing, sustainable agriculture and bioremediation applications^92–95^. For instance, an *Eschericia coli* co-culture was designed for bio-ethanol production using xylan. The co-operative co-culture resulted in higher yields, where the first strain was designed for secreting hemicellulases to break the substrate into sugars while the second was responsible for fermentation of these sugars into ethanol^96^. Another noteworthy application of SynCom is degradation of recalcitrant pollutants such as plastics. A recent study by Salinas et al. reported that microbial consortia of fungus; *Fusarium* and *Aspergillus* sp. Along with bacterial species i.e., *Bacillus* and *Pseudomonas* significantly degrade linear low-density polyethylene and other varieties of plastic^97^. Additionally, SynCom offers advantages over genetically modified monoculture for biochemical processes, in aspects such as metabolic resource allocation, fitness, redox balance and cell growth^98^. Such division of labor within microbial species was employed by Bao et al., for upcycling of polyethylene terephthalate using two engineered *Pseudomonas putida* strains to produce medium-chain length polyhydroxyalkanoate and muconic acid^99^. However, there exists significant challenges in employing SynCom, such as community stability, undesired microbial contamination, horizontal gene transfer within microbial populations and limited cell-cell communication^98^. Thus, optimizing the performance of SynComs requires a regulated environment, adapting tools like directed evolution and developing high-throughput screening methods for community-scale evaluation.

## Applications of DDSM in clean technology

At present, synthetic microbes are widely employed for sequestering GHG emissions, plastic and other waste remediation and biomanufacturing of alternative food and polymer. Data-driven synthetic microbes (DDSMs) are genetically engineered microbial strains that are designed and optimized using the knowledge obtained from multi-omics data, computational models, and simulations. These microbes are developed using a systems biology approach to predict and fine-tune their metabolic behavior for specific applications, ranging from bioremediation to sustainable bioproduction. In this article, we will discuss a few relevant examples where data-dependent synthetic microbes aid in attaining cleaner technology. As discussed previously, ML based protein engineering has improved the efficiency of PET degrading enzyme, PETase^41^. Additionally, for assimilation of PET monomers (ethylene glycol & terephthalate), *P. putida* KT2440 underwent an automation-enabled ALE on monomers for 350 generations^100^. In order to create better whole-cell biocatalysts for PET upcycling, an iterative strain engineering procedure that included heterologous pathway engineering, ALE, whole genome sequencing, and genome editing discovered five genetic interventions that promote *P. putida* growth on terephthalic acid. Aliphatic polyamides, widely used in textiles and car parts were also reported to be degraded by adapted strain of *P. putida* KT2440, that can metabolize 6-aminohexanoic acid, ε-caprolactam and 1,6-hexamethylenediamine^101^. The identified mutations in the adapted strains were found using whole genome sequencing, and several computational tools were used for optimization of synthetic constructs.

Polyhydroxyalkanoates (PHAs) are naturally occurring polymers that can be used as sustainable bioplastic. The PHA biosynthesis process is highly regulated by C/N ratio, specifically the synthesis is triggered by nitrogen limitations. Quantum-like models offer a creative way to describe complex, uncertain bacterial behavior such as modeling microbial decision-making under stress, that can help to improve bioproducts. For this purpose, a quantum modeling approach was used for optimization of PHA yields by varying C/N ratio is *P. putida*, with a maximum content of 13.81% cell dry mass at the C/N ratio of 40:1^101,102^. In another study, a computer aided Hub Metabolite-based Autoregulation system was used for conversion of lignin-derived aromatics (LDAs) to bioplastics (poly(3-hydroxybutyrate); PHB). This approach can improve metabolism efficiency and regulate the expression of functional genes in response to heterologous LDAs^103^. The strain’s stability and lignin conversion capabilities are further strengthened by multi-module genome integration and directed evolution, which results in a PHB production titer of 2.38 g L^-1^ while using heterologous LDAs as the only carbon source. In *Halomonas bluephagenesis*, fine-tuning of metabolic pathways resulted in enhanced production of PHB along with ectoine, a compatible solute^104^. Researchers have also developed a flexible, biodegradable and self-healing plastic like paper fabricated with protein nanofibers, using genetically engineered bacterial species^105^. Transcriptomic, metabolomic, and phylogenomic analysis played a key role in a recent breakthrough in waste to food transformation, which showed that *Neurospora intermedia* could grow on a variety of by-products, including fruit and vegetable pomace and plant-based milk waste, did not encode mycotoxins, and can be consumed as food alternative^106^.

## Challenges and limitations in DDSM

Although data-driven synthetic biology has revolutionary promise, a number of significant obstacles need to be overcome before DDSMs can be used in the real world. These range from overlooked practical realities of biosafety, regulation, and ecological risk to limitations in predictive modeling and ML fidelity. These challenges must be addressed by combining systems biology, robust ML techniques, and realistic constraints to create deployable and reliable DDSM systems. There is still a gap between in silico predictions and experimental results, even with advancements in computational modeling and omics integration. GSMMs frequently make assumptions that would not hold true in dynamic environmental situations, such as optimal growth or steady-state flux. Furthermore, especially for new enzymes or pathways with insufficient training data, ML models may overfit, be difficult to interpret, or predict rare phenotypes incorrectly^107^. The GSMMs were evaluated using MEMOTE, a standardized tool that provides a comprehensive quality score based on multiple metrics^108^. While MEMOTE scores offer useful insights, lower scores do not necessarily reflect poor model quality, as they may result from missing annotations rather than structural inaccuracies. To increase model accuracy, validation techniques such as iterative DBTL cycles, fluxomics, proteomics integration, and 13C-labeling experiments are essential. High-throughput mutant generation and phenotyping can offer one validation for GSMM. For this purpose, four *E. coli* models were tested for predictive accuracy using large-scale mutant fitness data across 25 carbon sources^109^. The key sources of error, including missing cofactors, incorrect assumptions about nutrient availability, and isoenzyme mapping inaccuracies were identified in the most recent model (*iML1515*). Additionally, ML algorithms identify key metabolic fluxes like hydrogen ion exchange, as influential to prediction accuracy. The work offers a framework for systematic GSMM validation while offering suggestions for improvement.

To prevent overfitting to merely functional data, it is crucial to add variation to training data in DDSMs, particularly by producing negative variants like nonsense mutations. Saturation mutagenesis and sequence shuffling are two methods that increase model variety. The model can be directed to the appropriate regions using truncation analysis and domain tools. Techniques like contrastive learning or adversarial training are helpful for handling uncommon or rare circumstances. Moreover, in order to increase reliability, models should incorporate confidence scores utilizing ensembles or Monte Carlo Dropout^110^. A number of crucial elements must be met for genetically modified organisms (GMOs) to be safely released into the environment and for them to be functional outside of lab settings: (1) constrained population activation and containment; (2) genetic stability of designed circuits; (3) reduction of resource-based interactions and cellular burden; and (4) reduction of variability due to population heterogeneity or external disruptions. These concerns and methods to sustain such challenges are reviewed by Kumar et al.^111^. To stop horizontal gene transfer or unintentional growth, DDSM chassis must incorporate confinement circuits, synthetic auxotrophy, and toxin-antitoxin kill switches. Frameworks for GMOs are in place, but there are supervision gaps for AI-designed enzymes, modular genomic constructions put together in silico, and SynComs. Furthermore, there is still a lack of adequate characterization of ecological concerns, such as alteration of the microbiome, gene flow to native species, or loss of function under stress. Embracing complexity and real-world context will help make data-driven synthetic biology a reliable tool for sustainable bioengineering.

## Conclusions

**Fig. 2:**
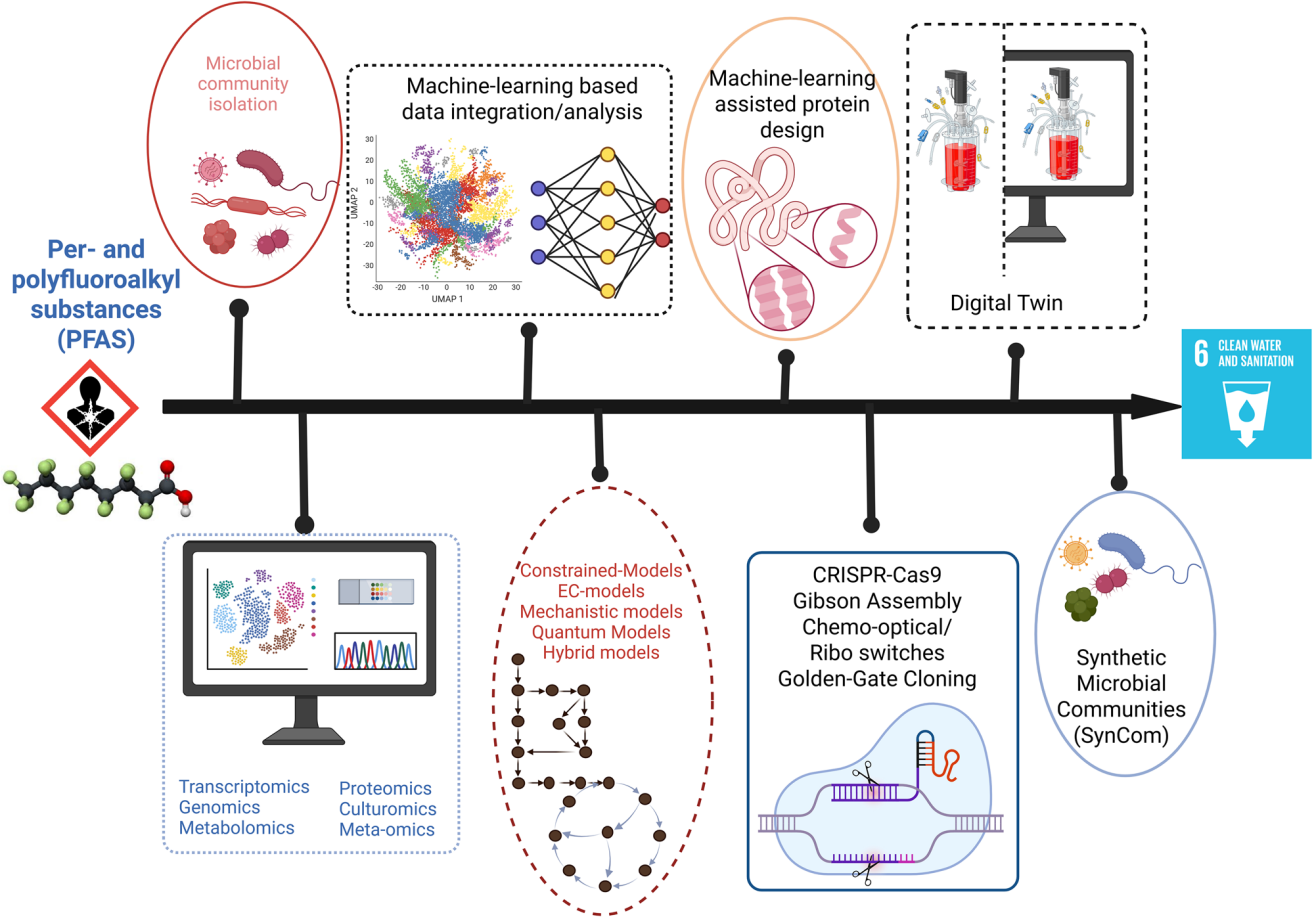
Framework for designing DDSM or SynCom to tackle forever chemicals (PFAS). The first step in designing DDSM requires generating large-scale experimental and omics datasets for microbial community isolated from contaminated area. This ‘big data’ is integrated using ML algorithms to extract important features. Additionally, this data can be transformed into a mathematical model, which can be simulated to predict desired phenotypic outcomes. Synthetic biology can leverage this model-guided metabolic information for creating in vitro genetic perturbations, crafting synthetic microbial cells or synthetic microbial communities (SynComs) offering promising sustainable solutions. Created in BioRender. Mariam, I. (2025) https://BioRender.com/kknl72a.

Leveraging big-data, computational science and automation, the field of synthetic biology has come a long way. The advancements in these data-dependent approaches have highly impacted microbial metabolism that span from designing new-to-nature enzymes or a complex biosynthetic pathway. Such DDSM has been widely implicated in degradation of recalcitrant pollutants, biomanufacturing of novel products and even engineered living materials. However, such big data and synthetic microbial technology has yet not been employed for degradation of forever chemicals and battery recycling, which is the need of the hour. Figure 2 illustrates a workflow that can be employed for bioremediation of PFAS using DDSM and SynComs. In this multi-step workflow, the first step is generating omics datasets for microbial populations in the PFAS-contaminated area (**Big Data**), analyzed using computational & ML approach (**Computational Tools to Decode Big Data**). The first two steps provide insights into metabolic pathways and enzymes responsible for degradation which will be further used for construction and simulation of metabolic models as described previously (**Defining Metabolic Blueprint)**. The participating enzymes can be rationally designed using ML algorithms (**Data-Driven Design of Synthetic Enzymes)** while the microbes can be synthetically modified using **advanced synthetic biology** tools. These insights feed into a **digital twin**, guiding the rational design of engineered microbes, further to overcome metabolic limitations and enhance degradation efficiency, DDSMs are configured into **SynComs**, enabling division of labor and adaptive synergy within microbial consortia. The engineered strains and communities undergo high-throughput testing, and their performance is iteratively optimized via the DBTL cycle, informed by dynamic in silico predictions. While the field has several challenges such as automated lab setup, unavailability of an intact digital twin process and lack of reproducibility during process scaling, that require immediate attention. Resolving these issues will hasten the creation of safe, scalable, and effective artificial microorganisms for use in environmental and industrial settings for clean technology.

## Data Availability

No datasets were generated or analyzed during the current study.

## References

[1] Pörtner, H.-O. et al. Technical Summary. In: Climate Change 2022: Impacts, Adaptation, and Vulnerability. Contribution of Working Group II to the Sixth Assessment Report of the Intergovernmental Panel on Climate Change [Pörtner, H.-O. et al. (eds.)]. 37–118 (Cambridge University Press, 2022).

[2] United Nations. *Transforming our world: the 2030 Agenda for Sustainable Developme*nt. A/RES/70/1. (United Nations, 2015).

[3] Newton, I. Philosophiæ Naturalis Principia Mathematica (Annotated by Nicolae Sfetcu). (Multidisciplinary Digital Publishing Institute, 2014).

[4] Timmis, K. et al. Microbes saving lives and reducing suffering. *Micro. Biotechnol.* **18**, e70068 (2025). 10.1111/1751-7915.70068 PMC11754571 39844583

[5] Timmis, K. et al. The contribution of microbial biotechnology to economic growth and employment creation. *Micro. Biotechnol.* **10**, 1137–1144 (2017). 10.1111/1751-7915.12845 PMC5609265 28868756

[6] LaCroix, R. A., Palsson, B. O. & Feist, A. M. A model for designing adaptive laboratory evolution experiments. *Appl. Environ. Microbiol.* **83**, e03115 (2017). 10.1128/AEM.03115-16 PMC5377496 28159796

[7] Jones, E. M., Marken, J. P. & Silver, P. A. Synthetic microbiology in sustainability applications. *Nat. Rev. Microbiol.* **22**, 345–359 (2024). 38253793 10.1038/s41579-023-01007-9

[8] Voigt, C. A. Synthetic biology 2020–2030: six commercially-available products that are changing our world. *Nat. Commun.* **11**, 1–6 (2020). 33311504 10.1038/s41467-020-20122-2 PMC7733420

[9] Himmelreich, R. et al. Complete sequence analysis of the genome of the bacterium Mycoplasma pneumoniae. *Nucleic Acids Res.* **24**, 4420 (1996). 8948633 10.1093/nar/24.22.4420 PMC146264

[10] Bukhman, Y. V. et al. A high-quality blue whale genome, segmental duplications, and historical demography. *Mol. Biol. Evol.* **41**, msae036 (2024). 38376487 10.1093/molbev/msae036 PMC10919930

[11] Westerhoff, H. V. & Palsson, B. O. The evolution of molecular biology into systems biology. *Nat. Biotechnol.* **22**, 1249–1252 (2004). 15470464 10.1038/nbt1020

[12] Wu, C. et al. A generalized computational framework to streamline thermodynamics and kinetics analysis of metabolic pathways. *Metab. Eng.* **57**, 140–150 (2020). 31401243 10.1016/j.ymben.2019.08.006

[13] Meesil, W. et al. Genome mining reveals novel biosynthetic gene clusters in entomopathogenic bacteria. *Sci. Rep.* **13**, 1–10 (2023). 38007490 10.1038/s41598-023-47121-9 PMC10676414

[14] Martin, L. B. B. et al. Complete biosynthesis of the potent vaccine adjuvant QS-21. *Nat. Chem. Biol.* **20**, 493–502 (2024). 38278997 10.1038/s41589-023-01538-5 PMC10972754

[15] Carbonell, P. et al. An automated Design-Build-Test-Learn pipeline for enhanced microbial production of fine chemicals. *Commun. Biol.* **1**, 66 (2018). 30271948 10.1038/s42003-018-0076-9 PMC6123781

[16] Sanger, F. et al. Nucleotide sequence of bacteriophage phi X174 DNA. *Nature* **265**, 687–695 (1977). 10.1038/265687a0 870828

[17] Fernández, P. et al. A 160 Gbp fork fern genome shatters size record for eukaryotes. *iScience* **27**, 109889 (2024). 39055604 10.1016/j.isci.2024.109889 PMC11270024

[18] Manzoni, C. et al. Genome, transcriptome and proteome: the rise of omics data and their integration in biomedical sciences. *Brief. Bioinforma.* **19**, 286 (2016). 10.1093/bib/bbw114 PMC6018996 27881428

[19] Big data in biology The hope and present-day challenges in it. *Gene Rep.* **21**, 100869 (2020).

[20] Database Commons A catalog of worldwide biological databases. *Genomics Proteom. Bioinforma.* **21**, 1054–1058 (2023). 10.1016/j.gpb.2022.12.004 PMC10928426 36572336

[21] Leinonen, R. et al. The European nucleotide archive. *Nucleic Acids Res.* **39**, D28 (2010). 20972220 10.1093/nar/gkq967 PMC3013801

[22] Rahuman, S. et al. Nicolas Rodriguez, Michael Hucka, and Henning Hermjakob BioModels - 15 years of sharing computational models in life science. *N**ucl. Acids Res*. **48**, 407–415 (2020). 10.1093/nar/gkz1055 PMC7145643 31701150

[23] Chetverikov, S. P., Sharipov, D. A., Korshunova, T. Y. & Loginov, O. N. Degradation of perfluorooctanyl sulfonate by strain Pseudomonas plecoglossicida 2.4-D. *Appl. Biochem. Microbiol.* **53**, 533–538 (2017).

[24] PFAS biodegradation by Labrys portucalensis F11 Evidence of chain shortening and identification of metabolites of PFOS, 6:2 FTS, and 5:3 FTCA. *Sci. Total Environ.* **959**, 178348 (2025). 39756302 10.1016/j.scitotenv.2024.178348 PMC13104705

[25] Dong, S. et al. Using network analysis and predictive functional analysis to explore the fluorotelomer biotransformation potential of soil microbial communities. *Environ. Sci. Technol.* **58**, 7480–7492 (2024). 38639388 10.1021/acs.est.4c00942

[26] Huang, Y. et al. High-throughput microbial culturomics using automation and machine learning. *Nat. Biotechnol.* **41**, 1424–1433 (2023). 36805559 10.1038/s41587-023-01674-2 PMC10567565

[27] Arango-Argoty, G. et al. DeepARG: a deep learning approach for predicting antibiotic resistance genes from metagenomic data. *Microbiome* **6**, 23 (2018). 29391044 10.1186/s40168-018-0401-z PMC5796597

[28] Li, Y. et al. HMD-ARG: hierarchical multi-task deep learning for annotating antibiotic resistance genes. *Microbiome* **9**, 40 (2021). 33557954 10.1186/s40168-021-01002-3 PMC7871585

[29] Johnson, M. et al. NCBI BLAST: a better web interface. *Nucleic Acids Res.* **36**, W5–W9 (2008). 18440982 10.1093/nar/gkn201 PMC2447716

[30] Xu, C. & Jackson, S. A. Machine learning and complex biological data. *Genome Biol.* **20**, 1–4 (2019). 30992073 10.1186/s13059-019-1689-0 PMC6469083

[31] Omics of oil biodegradation. *Curr. Opin. Chem. Eng.* **36**, 100800 (2022).

[32] Prasad, M. et al. *Alcanivorax borkumensis* biofilms enhance oil degradation by interfacial tubulation. *Science* **381**, 748–753 (2023). 37590351 10.1126/science.adf3345

[33] A novel bioprocess engineering approach to recycle hydrophilic and hydrophobic waste under high salinity conditions for the production of nutraceutical compounds. *Chem. Eng. J.* **431**, 133955 (2022).

[34] Mariam, I. et al. Transcriptomics aids in uncovering the metabolic shifts and molecular machinery of Schizochytrium limacinum during biotransformation of hydrophobic substrates to docosahexaenoic acid. *Micro. Cell Fact.* **23**, 97 (2024). 10.1186/s12934-024-02381-6 PMC10983653 38561811

[35] Yokoyama, D., Takamura, A., Tsuboi, Y. & Kikuchi, J. Large-scale omics dataset of polymer degradation provides robust interpretation for microbial niche and succession on different plastisphere. *ISME Commun.* **3**, 1–10 (2023). 37400632 10.1038/s43705-023-00275-z PMC10317964

[36] Dobson, L. et al. TmAlphaFold database: membrane localization and evaluation of AlphaFold2 predicted alpha-helical transmembrane protein structures. *Nucleic Acids Res.* **51**, D517–D522 (2023). 36318239 10.1093/nar/gkac928 PMC9825488

[37] Evans, R. et al. Protein complex prediction with alphafold-multimer. *B**ioinformatics* **13**, 6028 (2021).

[38] Notin, P., Rollins, N., Gal, Y., Sander, C. & Marks, D. Machine learning for functional protein design. *Nat. Biotechnol.* **42**, 216–228 (2024). 38361074 10.1038/s41587-024-02127-0 PMC13159571

[39] Hopf, T. A. et al. Mutation effects predicted from sequence co-variation. *Nat. Biotechnol.* **35**, 128–135 (2017). 28092658 10.1038/nbt.3769 PMC5383098

[40] Wu, Z., Kan, S. B. J., Lewis, R. D., Wittmann, B. J. & Arnold, F. H. Machine learning-assisted directed protein evolution with combinatorial libraries. *Proc. Natl. Acad. Sci. USA* **116**, 8852–8858 (2019). 30979809 10.1073/pnas.1901979116 PMC6500146

[41] Lu, H. et al. Machine learning-aided engineering of hydrolases for PET depolymerization. *Nature* **604**, 662–667 (2022). 35478237 10.1038/s41586-022-04599-z

[42] Lin, Z. et al. Evolutionary-scale prediction of atomic-level protein structure with a language model. *Science* **379**, 1123–1130 (2023). 36927031 10.1126/science.ade2574

[43] Repecka, D. et al. Expanding functional protein sequence spaces using generative adversarial networks. *Nat. Mach. Intell.* **3**, 324–333 (2021).

[44] Hawkins-Hooker, A. et al. Generating functional protein variants with variational autoencoders. *PLoS Comput. Biol.* **17**, e1008736 (2021). 33635868 10.1371/journal.pcbi.1008736 PMC7946179

[45] Zhou, G., Pang, Z., Lu, Y., Ewald, J. & Xia, J. OmicsNet 2.0: a web-based platform for multi-omics integration and network visual analytics. *Nucleic Acids Res.* **50**, W527–W533 (2022). 35639733 10.1093/nar/gkac376 PMC9252810

[46] Argelaguet, R. et al. Multi-omics factor analysis-a framework for unsupervised integration of multi-omics data sets. *Mol. Syst. Biol.* **14**, e8124 (2018). 29925568 10.15252/msb.20178124 PMC6010767

[47] Wang, B. et al. Similarity network fusion for aggregating data types on a genomic scale. *Nat. Methods* **11**, 333–337 (2014). 24464287 10.1038/nmeth.2810

[48] Lanckriet, G. R., De Bie, T., Cristianini, N., Jordan, M. I. & Noble, W. S. A statistical framework for genomic data fusion. *Bioinformatics* **20**, 2626–2635 (2004). 10.1093/bioinformatics/bth294 15130933

[49] Becker, S. A. et al. Quantitative prediction of cellular metabolism with constraint-based models: the COBRA Toolbox. *Nat. Protoc.* **2**, 727–738 (2007). 17406635 10.1038/nprot.2007.99

[50] Herrgard, M. J. Reconstruction and systems analysis of genome-scale metabolic and regulatory networks in saccharomyces cerevisiae. *Genome Res.* **13**, 244–253 (2004).

[51] Pathania, R. et al. Metabolic systems biology and multi-omics of cyanobacteria: perspectives and future directions. *Bioresour. Technol.* **343**, 126007 (2022). 34634665 10.1016/j.biortech.2021.126007

[52] Chen, Y. et al. Reconstruction, simulation and analysis of enzyme-constrained metabolic models using GECKO Toolbox 3.0. *Nat. Protoc.* **19**, 629–667 (2024). 38238583 10.1038/s41596-023-00931-7

[53] Aung, H. W., Henry, S. A. & Walker, L. P. Revising the Representation of Fatty Acid, Glycerolipid, and Glycerophospholipid Metabolism in the Consensus Model of Yeast Metabolism. *Ind. Biotechnol.* **9**, 215–228 (2013). 10.1089/ind.2013.0013 PMC3963290 24678285

[54] Ryu, J. Y., Kim, H. U. & Lee, S. Y. Deep learning enables high-quality and high-throughput prediction of enzyme commission numbers. *Proc. Natl Acad. Sci. USA* **116**, 13996–14001 (2019). 31221760 10.1073/pnas.1821905116 PMC6628820

[55] Li, F. et al. Deep learning-based kcat prediction enables improved enzyme-constrained model reconstruction. *Nat. Catal.* **5**, 662–672 (2022).

[56] Kroll, A., Rousset, Y., Hu, X.-P., Liebrand, N. A. & Lercher, M. J. Turnover number predictions for kinetically uncharacterized enzymes using machine and deep learning. *Nat. Commun.* **14**, 4139 (2023). 37438349 10.1038/s41467-023-39840-4 PMC10338564

[57] Wendering, P., Arend, M., Razaghi-Moghadam, Z. & Nikoloski, Z. Data integration across conditions improves turnover number estimates and metabolic predictions. *Nat. Commun.* **14**, 1485 (2023). 36932067 10.1038/s41467-023-37151-2 PMC10023748

[58] Niarakis, A. & Helikar, T. A practical guide to mechanistic systems modeling in biology using a logic-based approach. *Brief. Bioinform*. **22**, bbaa236 (2021). 10.1093/bib/bbaa236 PMC8293813 33064138

[59] Zhang, C. et al. Yeast9: a consensus genome-scale metabolic model for S. cerevisiae curated by the community. *Mol. Syst. Biol.* **20**, 1134–1150 (2024). 10.1038/s44320-024-00060-7 PMC11450192 39134886

[60] Simulation and optimization of dynamic flux balance analysis models using an interior point method reformulation. *Comp. Chem. Eng.* **119**, 152–170 (2018).

[61] Kim, J. & Reed, J. L. OptORF: optimal metabolic and regulatory perturbations for metabolic engineering of microbial strains. *BMC Syst. Biol.* **4**, 53 (2010). 20426856 10.1186/1752-0509-4-53 PMC2887412

[62] Burgard, A. P., Pharkya, P. & Maranas, C. D. Optknock: a bilevel programming framework for identifying gene knockout strategies for microbial strain optimization. *Biotechnol. Bioeng.* **84**, 647–657 (2003). 14595777 10.1002/bit.10803

[63] Ranganathan, S., Suthers, P. F. & Maranas, C. D. OptForce: an optimization procedure for identifying all genetic manipulations leading to targeted overproductions. *PLoS Comput. Biol.* **6**, e1000744 (2010). 20419153 10.1371/journal.pcbi.1000744 PMC2855329

[64] Tepper, N. & Shlomi, T. Predicting metabolic engineering knockout strategies for chemical production: accounting for competing pathways. *Bioinformatics* **26**, 536–543 (2010). 20031969 10.1093/bioinformatics/btp704

[65] Segrè, D., Vitkup, D. & Church, G. M. Analysis of optimality in natural and perturbed metabolic networks. *Proc. Natl. Acad. Sci. USA* **99**, 15112–15117 (2002). 12415116 10.1073/pnas.232349399 PMC137552

[66] Kim, J. & Reed, J. L. RELATCH: relative optimality in metabolic networks explains robust metabolic and regulatory responses to perturbations. *Genome Biol.* **13**, R78 (2012). 23013597 10.1186/gb-2012-13-9-r78 PMC3506949

[67] Shlomi, T., Berkman, O. & Ruppin, E. Regulatory on/off minimization of metabolic flux changes after genetic perturbations. *Proc. Natl. Acad. Sci. USA* **102**, 7695–7700 (2005). 15897462 10.1073/pnas.0406346102 PMC1140402

[68] Czajka, J. J. et al. FluxRETAP: A REaction TArget Prioritization Genome-Scale Modeling Technique for Selecting Genetic Targets. 10.21203/rs.3.rs-5961146/v1 (2025). 10.1093/bioinformatics/btaf471 PMC12417073 40848245

[69] Doudna, J. A. & Charpentier, E. Genome editing. The new frontier of genome engineering with CRISPR-Cas9. *Science* **346**, 1258096 (2014). 25430774 10.1126/science.1258096

[70] Shah, K., Kaur, A., Saxena, S. & Arora, S. CRISPR-based approach: A way forward to sustainable development goals (SDGs). In *Gene Editing in Plants* 709–733 (Springer Nature, 2024).

[71] Matinvafa, M. A. et al. CRISPR-Cas technology secures sustainability through its applications: a review in green biotechnology. *3 Biotech* **13**, 383 (2023). 37920190 10.1007/s13205-023-03786-7 PMC10618153

[72] Wang, S.-W. et al. Current applications and future perspective of CRISPR/Cas9 gene editing in cancer. *Mol. Cancer* **21**, 57 (2022). 35189910 10.1186/s12943-022-01518-8 PMC8862238

[73] Khoshandam, M., Soltaninejad, H., Mousazadeh, M., Hamidieh, A. A. & Hosseinkhani, S. Clinical applications of the CRISPR/Cas9 genome-editing system: Delivery options and challenges in precision medicine. *Genes Dis.* **11**, 268–282 (2024). 37588217 10.1016/j.gendis.2023.02.027 PMC10425811

[74] Pacesa, M., Pelea, O. & Jinek, M. Past, present, and future of CRISPR genome editing technologies. *Cell* **187**, 1076–1100 (2024). 38428389 10.1016/j.cell.2024.01.042

[75] Labun, K. et al. CHOPCHOP v3: expanding the CRISPR web toolbox beyond genome editing. *Nucleic Acids Res*. **47**, W171–W174 (2019). 31106371 10.1093/nar/gkz365 PMC6602426

[76] Weber, E., Engler, C., Gruetzner, R., Werner, S. & Marillonnet, S. A modular cloning system for standardized assembly of multigene constructs. *PLoS One* **6**, e16765 (2011). 21364738 10.1371/journal.pone.0016765 PMC3041749

[77] Engler, C., Kandzia, R. & Marillonnet, S. A one pot, one step, precision cloning method with high throughput capability. *PLoS One* **3**, e3647 (2008). 18985154 10.1371/journal.pone.0003647 PMC2574415

[78] Pereira, F. et al. Pydna: a simulation and documentation tool for DNA assembly strategies using python. *BMC Bioinformatics* **16**, 142 (2015). 10.1186/s12859-015-0544-x PMC4472420 25933606

[79] Gibson, D. G. et al. Enzymatic assembly of DNA molecules up to several hundred kilobases. *Nat. Methods* **6**, 343–345 (2009). 19363495 10.1038/nmeth.1318

[80] Angenent-Mari, N. M., Garruss, A. S., Soenksen, L. R., Church, G. & Collins, J. J. A deep learning approach to programmable RNA switches. *Nat. Commun.* **11**, 1–12 (2020). 33028812 10.1038/s41467-020-18677-1 PMC7541447

[81] Deep learning for optimization of protein expression. *Curr. Opin. Biotechnol.* **81**, 102941 (2023). 10.1016/j.copbio.2023.102941 37087839

[82] Fallahpour, A., Gureghian, V., Filion, G. J., Lindner, A. B. & Pandi, A. CodonTransformer: a multispecies codon optimizer using context-aware neural networks. *Nat. Commun.* **16**, 3205 (2025). 40180930 10.1038/s41467-025-58588-7 PMC11968976

[83] Salvail, H. & Breaker, R. R. Riboswitches. *Curr. Biol.* **33**, R343–R348 (2023). 37160088 10.1016/j.cub.2023.03.069 PMC11207198

[84] Jafarbeglou, F. & Dunlop, M. J. Red light responsive Cre recombinase for bacterial optogenetics. *ACS Synth. Biol.* **13**, 3991–4001 (2024). 39558834 10.1021/acssynbio.4c00388

[85] Carbonell, P., Radivojevic, T. & García Martín, H. Opportunities at the Intersection of Synthetic Biology, Machine Learning, and Automation. *ACS Synth. Biol.* **8**, 1474–1477 (2019). 31319671 10.1021/acssynbio.8b00540

[86] Gupta, S. D*igital Twins: Advancements in Theory, Implementation, and Applications*. (Springer Nature).

[87] Can digital twin efforts shape microorganism-based alternative food? *Curr. Opin. Biotechnol.* **87**, 103115 (2024). 10.1016/j.copbio.2024.103115 38547588

[88] Konur, S. et al. Toward full-stack synthetic biology: integrating model specification, simulation, verification, and biological compilation. *ACS Synth. Biol.* **10**, 1931–1945 (2021). 34339602 10.1021/acssynbio.1c00143

[89] Stephenson, A. et al. Physical laboratory automation in synthetic biology. *ACS Synth. Biol.* **12**, 3156–3169 (2023). 37935025 10.1021/acssynbio.3c00345

[90] New high throughput cell-selection system to speed medical advances and research. T*he University of Edinburgh*.

[91] McCarty, N. S. & Ledesma-Amaro, R. Synthetic biology tools to engineer microbial communities for biotechnology. *Trends Biotechnol.* **37**, 181–197 (2019). 30497870 10.1016/j.tibtech.2018.11.002 PMC6340809

[92] Brenner, K., You, L. & Arnold, F. H. Engineering microbial consortia: a new frontier in synthetic biology. *Trends Biotechnol.* **26**, 483–489 (2008). 18675483 10.1016/j.tibtech.2008.05.004

[93] Shayanthan, A., Ordoñez, P. A. C. & Oresnik, I. J. The role of synthetic microbial communities (SynCom) in sustainable agriculture. *Front. Agron*. **4**, 896307 (2022).

[94] Grosskopf, T. & Soyer, O. S. Synthetic microbial communities. *Curr. Opin. Microbiol.* **18**, 72–77 (2014). 24632350 10.1016/j.mib.2014.02.002 PMC4005913

[95] Shong, J., Jimenez Diaz, M. R. & Collins, C. H. Towards synthetic microbial consortia for bioprocessing. *Curr. Opin. Biotechnol.* **23**, 798–802 (2012). 22387100 10.1016/j.copbio.2012.02.001

[96] Shin, H.-D., McClendon, S., Vo, T. & Chen, R. R. Escherichia coli binary culture engineered for direct fermentation of hemicellulose to a biofuel. *Appl. Environ. Microbiol.* **76**, 8150–8159 (2010). 20935118 10.1128/AEM.00908-10 PMC3008245

[97] Salinas, J. et al. Construction of versatile plastic-degrading microbial consortia based on ligninolytic microorganisms associated with agricultural waste composting. *Environ. Pollut.* **366**, 125333 (2025). 39615570 10.1016/j.envpol.2024.125333

[98] Che, S. & Men, Y. Synthetic microbial consortia for biosynthesis and biodegradation: promises and challenges. *J. Ind. Microbiol. Biotechnol.* **46**, 1343–1358 (2019). 31278525 10.1007/s10295-019-02211-4

[99] Bao, T., Qian, Y., Xin, Y., Collins, J. J. & Lu, T. Engineering microbial division of labor for plastic upcycling. *Nat. Commun.* **14**, 5712 (2023). 37752119 10.1038/s41467-023-40777-x PMC10522701

[100] Werner, A. Z. et al. Adaptive laboratory evolution and genetic engineering improved terephthalate utilization in Pseudomonas putida KT2440. *Metab. Eng.* **88**, 196–205 (2024). 39701409 10.1016/j.ymben.2024.12.006

[101] de Witt, J. et al. Upcycling of polyamides through chemical hydrolysis and engineered Pseudomonas putida. *Nat. Microbiol.* **10**, 667–680 (2025). 10.1038/s41564-025-01929-5 PMC11879879 39929973

[102] Ho, L. Y. L. et al. Quantum modeling simulates nutrient effect of bioplastic polyhydroxyalkanoate (PHA) production in Pseudomonas putida. *Sci. Rep.* **14**, 1–9 (2024). 39107357 10.1038/s41598-024-68727-7 PMC11303679

[103] Zhao, Y. et al. Lignin valorization to bioplastics with an aromatic hub metabolite-based autoregulation system. *Nat. Commun.* **15**, 1–17 (2024). 39468081 10.1038/s41467-024-53609-3 PMC11519575

[104] Ma, H. et al. Rational flux-tuning of Halomonas bluephagenesis for co-production of bioplastic PHB and ectoine. *Nat. Commun.* **11**, 1–12 (2020). 32620759 10.1038/s41467-020-17223-3 PMC7334215

[105] Manjula-Basavanna, A., Duraj-Thatte, A. M. & Joshi, N. S. Mechanically Tunable, Compostable, Healable and Scalable Engineered Living Materials. *Nat. Commun.* **15**, 1–11 (2024). 39532836 10.1038/s41467-024-53052-4 PMC11557937

[106] Maini Rekdal, V. et al. Neurospora intermedia from a traditional fermented food enables waste-to-food conversion. *Nat. Microbiol.* **9**, 2666–2683 (2024). 39209985 10.1038/s41564-024-01799-3 PMC11445060

[107] Shi, Z. et al. Data-driven synthetic cell factories development for industrial biomanufacturing. *Biodes. Res.* **2022**, 9898461 (2022). 37850146 10.34133/2022/9898461 PMC10521697

[108] Lieven, C. et al. MEMOTE for standardized genome-scale metabolic model testing. *Nat. Biotechnol.* **38**, 272–276 (2020). 32123384 10.1038/s41587-020-0446-y PMC7082222

[109] Bernstein, D. B., Akkas, B., Price, M. N. & Arkin, A. P. Evaluating E. coli genome-scale metabolic model accuracy with high-throughput mutant fitness data. *Mol. Syst. Biol.* **19**, e11566 (2023). 37888487 10.15252/msb.202311566 PMC10698504

[110] Contreras, J. & Bocklitz, T. Enhancing decision confidence in AI using Monte Carlo dropout for Raman spectra classification. *Anal. Chim. Acta* **1332**, 343346 (2024). 39580162 10.1016/j.aca.2024.343346

[111] Kumar, S. & Hasty, J. Stability, robustness, and containment: preparing synthetic biology for real-world deployment. *Curr. Opin. Biotechnol.* **79**, 102880 (2023). 36621221 10.1016/j.copbio.2022.102880 PMC11623912

